# New technique for detecting cracked teeth and evaluating the crack depth by contrast-enhanced cone beam computed tomography: an in vitro study

**DOI:** 10.1186/s12903-022-02085-6

**Published:** 2022-03-02

**Authors:** Jie Zhou, Jieni Fu, Mo Xiao, Feng Qiao, Tiantian Fu, Yangyang Lv, Fei Wu, Cuicui Sun, Peng Li, Ligeng Wu

**Affiliations:** 1 Department of Stomatology, Wuqing People Hospital, Tianjin, China; 2 Department of Endodontics, Hangzhou Stomatological Hospital, Hangzhou, Zhejiang China; 3 Department of Endodontics and Restorative Dentistry, School of Stomatology, Tianjin Medical University, #12 Qi Xiang Tai Road, He Ping District, Tianjin, 300070 China; 4 Department of Oral and Maxillofacial Surgery, School of Stomatology, Tianjin Medical University, Tianjin, China; 5 Department of Endodontics, Wuxi Stomatology Hospital, Jiangsu, China; 6 Department of Endodontics, Yantai Stomatological Hospital Affiliated to Binzhou Medical College, Yantai, China; 7 Department of Endodontics, Tianjin Stomatological Hospital, Tianjin, China; 8 Department of Radiology, School of Stomatology, Tianjin Medical University, Tianjin, China

**Keywords:** Cracked teeth, Cone beam computed tomography, Micro-computed tomography, Periapical radiography, Crack depths, Ioversol

## Abstract

**Background:**

Cracked teeth may cause various clinical symptoms depending on the extension depth of the crack and the subsequent bacterial infections. However, techniques to reliably determine the extension depths of cracks in teeth before treatment are lacking. The aim of this study was to develop a new technique based on contrast-enhanced cone beam computed tomography (CBCT) to improve the accuracy of crack depth evaluation in vitro.

**Methods:**

We developed an in vitro artificial simulation model of cracked teeth. Pre-experimental CBCT (pre-CBCT), and micro-computed tomography (micro-CT) were first performed for all cracked teeth (n = 31). Contrast-enhanced CBCT was then performed by infiltrating the crack with ioversol under vacuum conditions. The sensitivities of pre-CBCT and contrast-enhanced CBCT for the diagnosis of cracked teeth were calculated. According to the K-means clusters, crack depths measured by micro-CT were changed into categorical variables. Bland–Altman plot and the intraclass correlation coefficient (ICC) were used to analyze the consistency of the crack depths between the pre-CBCT and contrast-enhanced CBCT, as well as the ICC between the contrast-enhanced CBCT and micro-CT. Receiver operating characteristic (ROC) curves were generated to assess the ability for predicting crack depth in the differential diagnosis using pre-CBCT and contrast-enhanced CBCT. Restricted cubic splines were also used to model the non-linear relationship between the crack depths of contrast-enhanced CBCT and micro-CT.

**Results:**

The sensitivities of pre-CBCT and contrast-enhanced CBCT were 48.4%, and 67.7%, respectively. The ICC value of crack depth as measured by pre-CBCT and contrast-enhanced CBCT was 0.847 (95% confidence interval [CI] 0.380–0.960; *P* < 0.001). The areas under ROC curves (AUC) of pre-CBCT and contrast-enhanced CBCT were different: the AUC of pre-CBCT was 0.958 (*P* = 0.000, 95% CI 0.843–1.074), and the AUC of contrast-enhanced CBCT was 0.979 (*P* = 0.000, 95% CI 0.921–1.037), and the difference was not statistically significant (Z = − 0.707, *P* = 0.480). The ICC value of crack depth as measured by contrast-enhanced CBCT and micro-CT was 0.753 (95% CI 0.248–0.911; *P* < 0.001).

**Conclusion:**

Contrast-enhanced CBCT under vacuum conditions with a contrast medium can significantly improve the crack detection rate of cracked teeth; however, it cannot measure the crack depths accurately.

## Background

Tooth cracks extend from the crown toward the apex and gradually propagate into the dentin, involving one or two marginal ridges [1, 2]. Cracked teeth may produce variable clinical symptoms depending on the extension depth of the crack and subsequent bacterial infections [3]. The typical symptoms are unexplained pain when exposed to a cold stimulus and sharp pain during chewing or releasing the occlusion [4]. Sometimes, a narrow and deep periodontal pocket indicates that the crack extends subgingivally [5, 6].

Several auxiliary approaches for diagnosing cracked teeth exist, including operating microscopes [7, 8], transillumination [9–11], methylene blue staining [9],and the Tooth Slooth [12]. In addition, quantitative percussion diagnosis [13] and direct supra-coronal splinting [14] have proven to be equally effective in a clinical setting. However, techniques to reliably determine the extension depths of cracked teeth before treatment are lacking.

Several studies have investigated the diagnosis and depth assessment of cracked teeth in vitro. Imai et al. [15–19] reported that optical coherence tomography (OCT) was effective in detecting cracks. OCT could effectively detect enamel cracks within 3 mm of depth but could not diagnose structural cracks that caused clinical symptoms. Jun et al. [20, 21] were the first to use quantitative laser-induced fluorescence (QLF) to detect tooth cracks; however, QLF could only diagnose enamel cracks. Matsushita–Tokugawa [22] used infrared thermography to detect cracked teeth. This technique could only detect cracks with a width of 4–35.5 μm; however, its performance was limited when the width exceeded 42 μm.

Radiological examination, such as cone beam computed tomography (CBCT), is a commonly used diagnostic tool. However, it can only be used to diagnose wide cracks [23, 24]. Additionally, this tool is usually applied to diagnose vertical root fractures (VRFs) [25–28]. A few studies have reported the efficiency of magnetic resonance imaging (MRI) in diagnosing cracked teeth; however, the reliability of MRI in vivo has not been confirmed [29, 30]. Micro-computed tomography (micro-CT) has high resolution and is commonly considered the gold standard diagnostic approach for detecting microcracks in vitro [31]. An in vitro study confirmed that infiltrating the crack with the contrast agent barium sulfate followed by contrast-enhanced micro-CT can produce high-density linear images of fine cracks on radiographs [32]. Therefore, the detection of fine cracks can be improved using micro-CT, and the crack invasion depth can be calculated; however, it can only be used in ex vivo experiments. Although the resolution of CBCT is not as good as that of micro-CT, it can be used in clinical practice; hence, the diagnostic applicability of CBCT in clinical settings needs to be improved.

In this study, to improve the effectiveness of CBCT as a tool for determining the crack depth of cracked teeth before treatment, we aimed to explore a technique based on contrast-enhanced CBCT, and investigated whether it would predict the crack depth of cracked teeth.

## Methods

From January to December 2018, we collected 200 intact teeth extracted using minimally invasive techniques for periodontal or orthodontic reasons at the Department of Oral and Maxillofacial Surgery, Stomatological Hospital of Tianjin Medical University. This study was approved by the ethics committee of the Stomatological Hospital of Tianjin Medical University (TMUhMEC2019014). The inclusion criterion was the absence of enamel cracks that were visible to the naked eye. The exclusion criteria were the presence of dental caries, wedge-shaped defects, and VRFs, and a previous history of root canal therapy or restoration. Finally, 40 teeth that met the criteria were screened in vitro.

An in vitro artificial simulation cracked tooth model was established. All the 40 screened intact teeth were cleaned to remove soft tissue and calculus, and soaked in 0.1% thymol solution for 24 h. Subsequently, they were placed in 0.9% saline solution, soaked in liquid nitrogen (− 196 °C) for 1 min and immediately transferred into hot water (90 °C) for 5 min [23, 33]. The teeth were then observed under a surgical operating microscope (OMS2350; Zumax, Suzhou, China) under 17 × magnification. If there were visible cracks, the teeth were regarded as artificially cracked. Among the 40 teeth, 31 were selected as artificially cracked; the remaining 9 teeth were excluded due to splits or absence of cracks. The 31 cracked teeth were randomly embedded in four trays, and the root portion of each tooth was wrapped in modeling wax to simulate the periodontal tissue.

The pre-CBCT (KaVo 3D eXam, Germany) parameters were as follows: voltage, 120 kV; current, 8 mA; matrix size, 640 × 640; field of view, 8 × 8 cm; and pixel size, 0.125 mm × 0.125 mm. To ensure better pre-CBCT imaging quality, the operations were performed by a technician. The pre-CBCT results were stored in a database, and pre-CBCT images were analyzed using the Vision Q (KaVo eXam Vision) software package.

The micro-CT (SIEMENS, Munich, Germany) parameters were as follows: voltage, 80 kV; current, 500 μA; resolution, 9.08 μm; exposure time, 1000 ms. The Inveon Research Workplace software (SIEMENS, Munich, Germany) was used to reconstruct three-dimensional (3D) images. The presence of cracks and their extension depths, as measured from the micro-CT images, was recorded by a radiologist who was blinded to the evaluation of the pre-CBCT images.

**Fig. 1:**
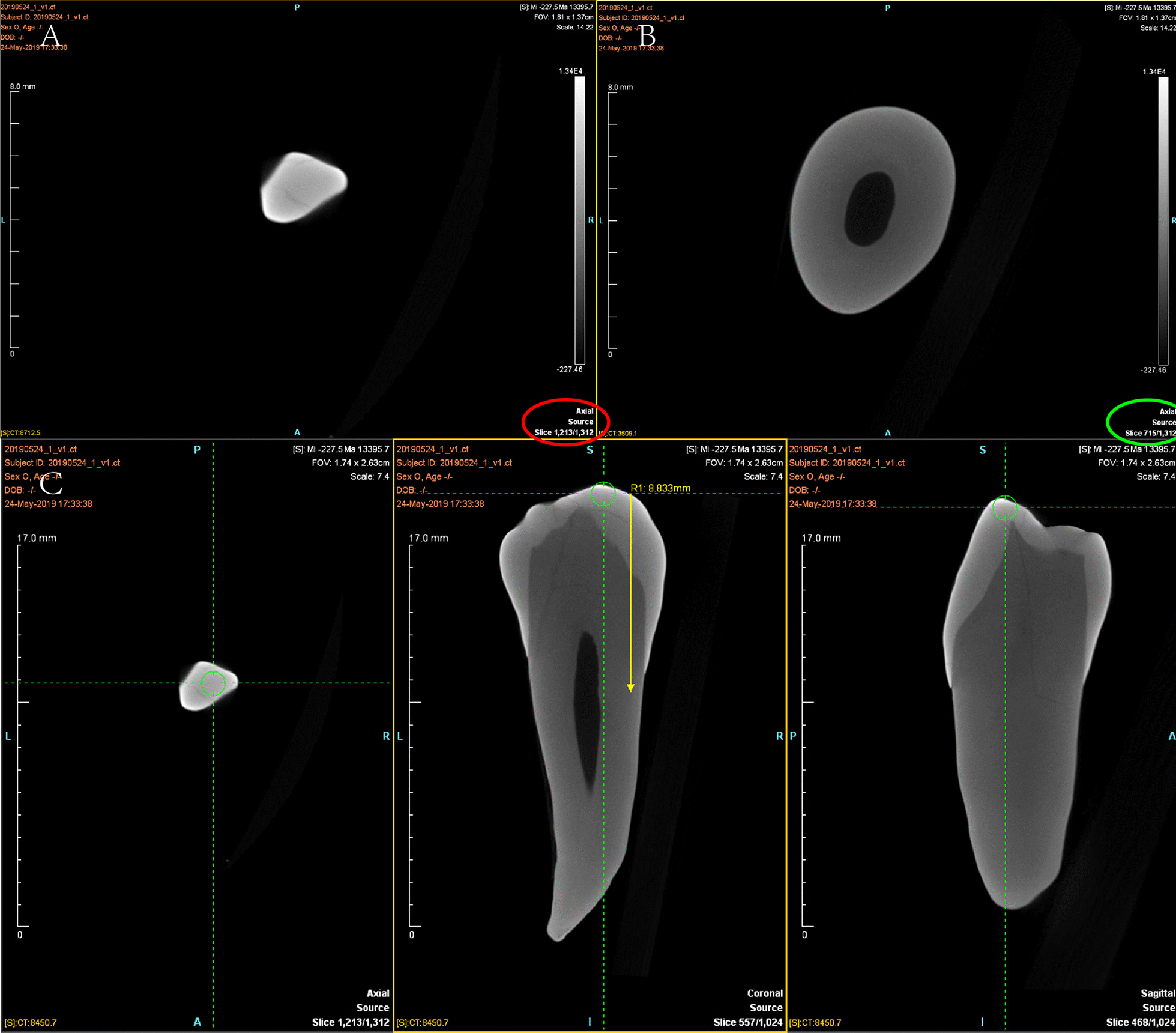
Measurement of the crack depth of a cracked tooth on micro-computed tomography. **A** Appearance of the crown crack on the horizontal plane at layer 1213 (indicated with a red circle); **B** complete disappearance of the root crack at layer 715 (indicated with a green circle); **C** the distance from layer 1213 to layer 715, as measured with the ruler tool (indicated with a yellow line), is the crack depth; in this case, the depth is 8.833 mm

The procedure to measure crack depth extending from the crown to the root on micro-CT images (Fig. 1A–C) was as follows: The cracked tooth was adjusted to an upright position in the coronal and sagittal planes and the cracks were observed from the crown to the root in the horizontal plane. When the crack appeared in the crown for the first time on the horizontal plane, the layer was recorded as “a”; the crack was observed extending to the root, always on the horizontal plane. When the crack began to disappear on the root, the layer was recorded as “b”; the distance from “a” to “b,” measured with the ruler tool, was considered to be the crack depth, not the actual crack length.

**Fig. 2:**
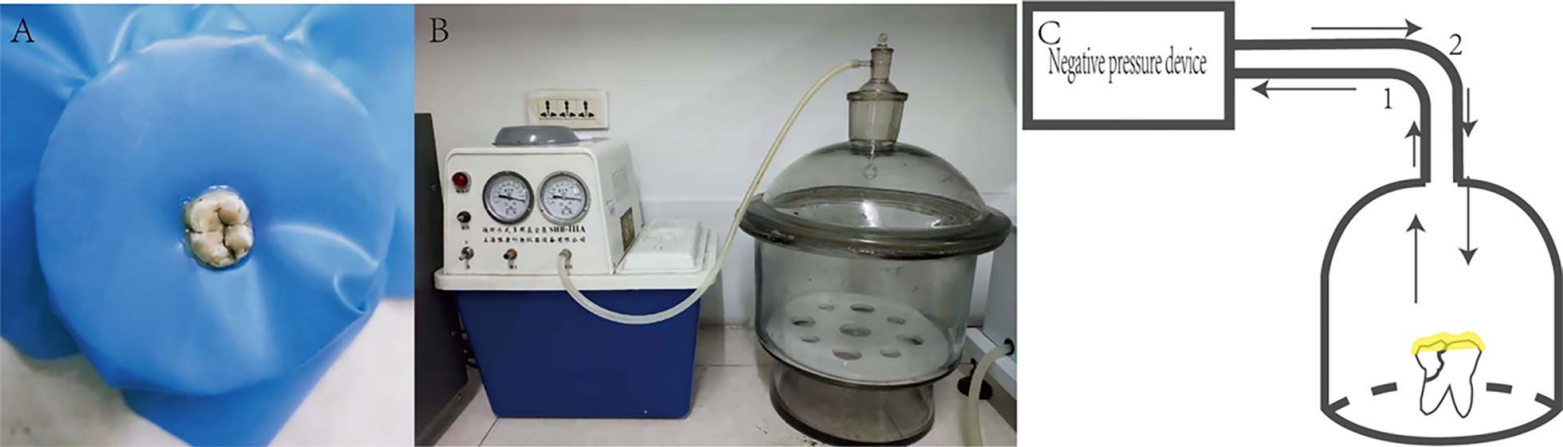
The schematic and experimental model system used to infiltrate the contrast medium into the crack. **A** The crown of the cracked tooth is exposed with a rubber dam. **B** The closed glass jar and suction pump device are connected to provide negative pressure suction. **C** Preparation on the occlusal surface with ioversol solution (indicated with yellow); 1: Negative pressure was applied gradually until it reached − 0.08 MPa and it was maintained at this value for 1 min; 2: Subsequently, normal atmospheric pressure was restored

Ioversol solution (C_18_H_24_I_3_N_3_O_9_; Hengrui Pharmaceutical, Jiangsu, China) was diluted in a ratio of 3:1 in normal saline to increase its fluidity. The crown and root of each cracked tooth were isolated with a rubber dam, and the crown was filled with the diluted ioversol solution. The teeth and the rubber dam were kept in place using a rubber band and a paper cup (Fig. 2A). The teeth were placed in a closed glass jar, connected to the suction pump with a rubber tube (Fig. 2B). The crack was infiltrated with ioversol under vacuum conditions (Fig. 2C). This procedure was repeated once again and the teeth were then removed from the jar. After infiltration, the cracked teeth remained in the trays with wax and underwent contrast-enhanced CBCT with the same parameters as used for pre-CBCT.

Two observers, an endodontics graduate student and an experienced radiologist, were involved in the evaluation. Before the study, the two observers were trained to achieve a high degree of consistency. Thereafter, the presence and absence of cracks were determined using pre-CBCT and contrast-enhanced CBCT by the two observers. In the event of disagreement between the observers, the image was re-examined until a consensus was reached. The following 2-point rating scale was used to determine whether cracks were present on pre-CBCT: (i) probably or definitely not a lesion, (ii) probably or definitely a lesion.

**Fig. 3:**
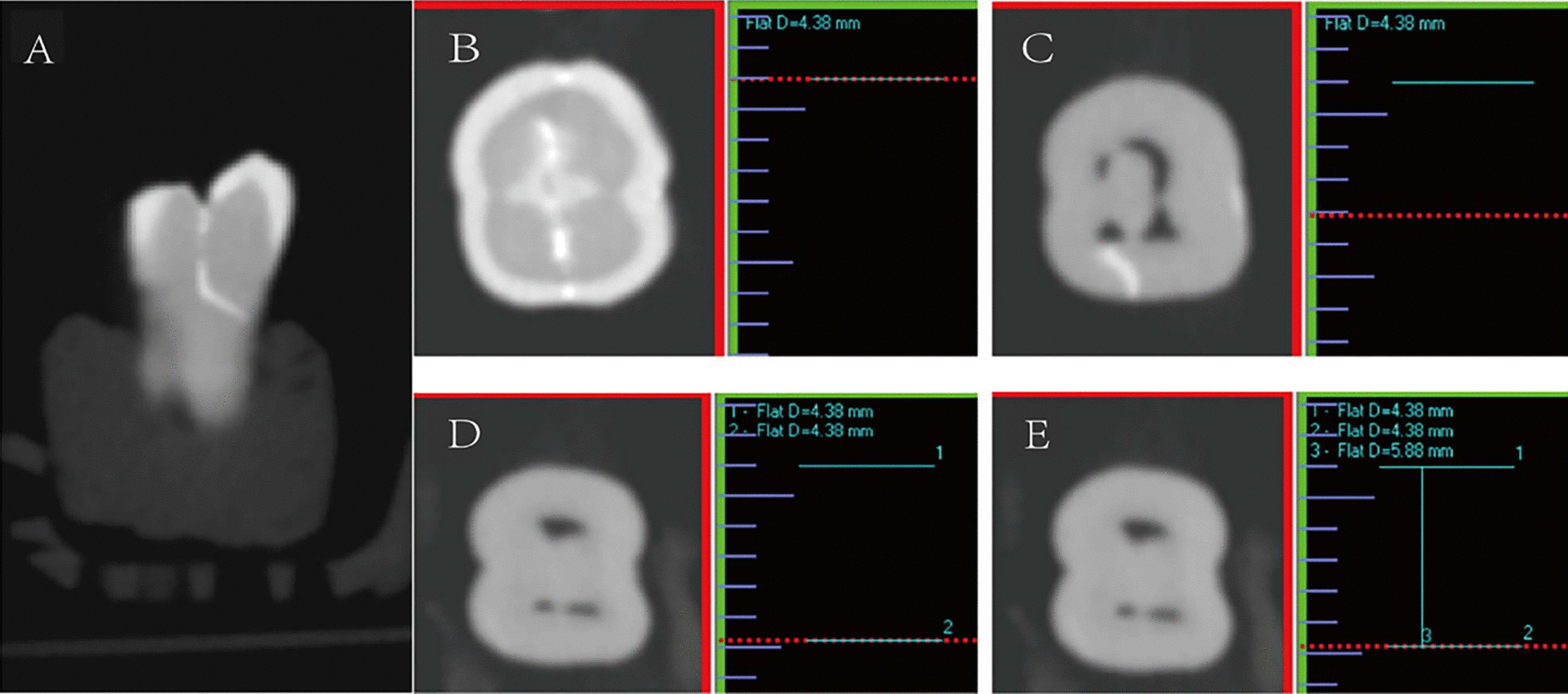
Method of measuring the crack depth of tooth no. 23 on contrast-enhanced cone beam computed tomography (contrast-enhanced CBCT). **A** The coronal plane of the crack shows the path of the crack. **B** The first location where the high-density crack started to appear on the contrast-enhanced CBCT scan is marked “1” in the horizontal plane. **C** A high-density crack image of a cracked tooth. **D** The crack completely disappeared and is marked “2”. **E** The distance from “1” to “2”,i.e., the length of segment “3”, was taken as the extension depth of the crack

On contrast-enhanced CBCT, a high-density linear crack was considered to mark a cracked tooth. The method used for measuring cracks on contrast-enhanced CBCT was the same as that used for micro-CT: the position of the tooth was adjusted in the sagittal and coronal planes to set it upright; the coronal plane of the crack shows its path (Fig. 3A); the cracks were observed from the crown to the root on the horizontal plane. When the high-density crack appeared in the crown for the first time on the horizontal plane, a horizontal line was marked as “1” in the coronal plane with the "distance" tool, indicating the level of the first occurrence of the crack (Fig. 3B). The crack extending to the root was always observed in the horizontal plane (Fig. 3C). When the high-density crack disappeared completely, the layer was marked as “2” in the coronal plane and represented the apical point of the crack (Fig. 3D). The "distance" tool was used to draw a vertical line from 1 to 2 in the coronal plane, and was marked as “3,” representing the crack depth as measured by contrast-enhanced CBCT (Fig. 3E). The extension depth of the cracked teeth on the contrast-enhanced CBCT was measured by the radiologist. Each crack depth value was measured three times at one-week intervals by the same radiologist, and the average value was considered as the final crack depth.

SPSS v.26.0 software (IBM Corp, Somers, NY), R software, version 3.6.0 (R Foundation for Statistical Computing; http://www.r-project.org/), and RStudio 1.1.463 (RStudio, PBC, Boston, MA, US) were used to perform the statistical analysis for the study.

The K-means clustering algorithm, first published in 1955, has been proposed over 50 years ago and is still widely used even though thousands of clustering algorithms have been published ever since [34]. Moreover, it is undoubtedly the most widely used partitional clustering algorithm [35]. Crack depths measured by micro-CT were transformed into categorical variables according to the K-means clusters.

For further analysis, the Bland–Altman plot was used to analyze the consistency of the crack depths between pre-CBCT and contrast-enhanced CBCT. The intraclass correlation coefficient (ICC) was used to evaluate the consistency of the crack depths as measured on pre-CBCT and contrast-enhanced CBCT, as well as between contrast-enhanced CBCT and micro-CT. The cracked teeth that could be detected by pre-CBCT and contrast-enhanced CBCT were included in the receiver operating characteristic (ROC) analysis. The crack depth of pre-CBCT and contrast-enhanced CBCT were continuous variables, and the crack depth of micro-CT was changed into a binary variable to study the predictive ability of pre-CBCT and contrast-enhanced CBCT for crack depth.

Moreover, we used restricted cubic splines (RCS) with 4 knots at the 5th, 35th, 65th, and 95th percentiles to model the non-linear relationship between the crack depths of contrast-enhanced CBCT and micro-CT.

## Results

**Fig. 4:**
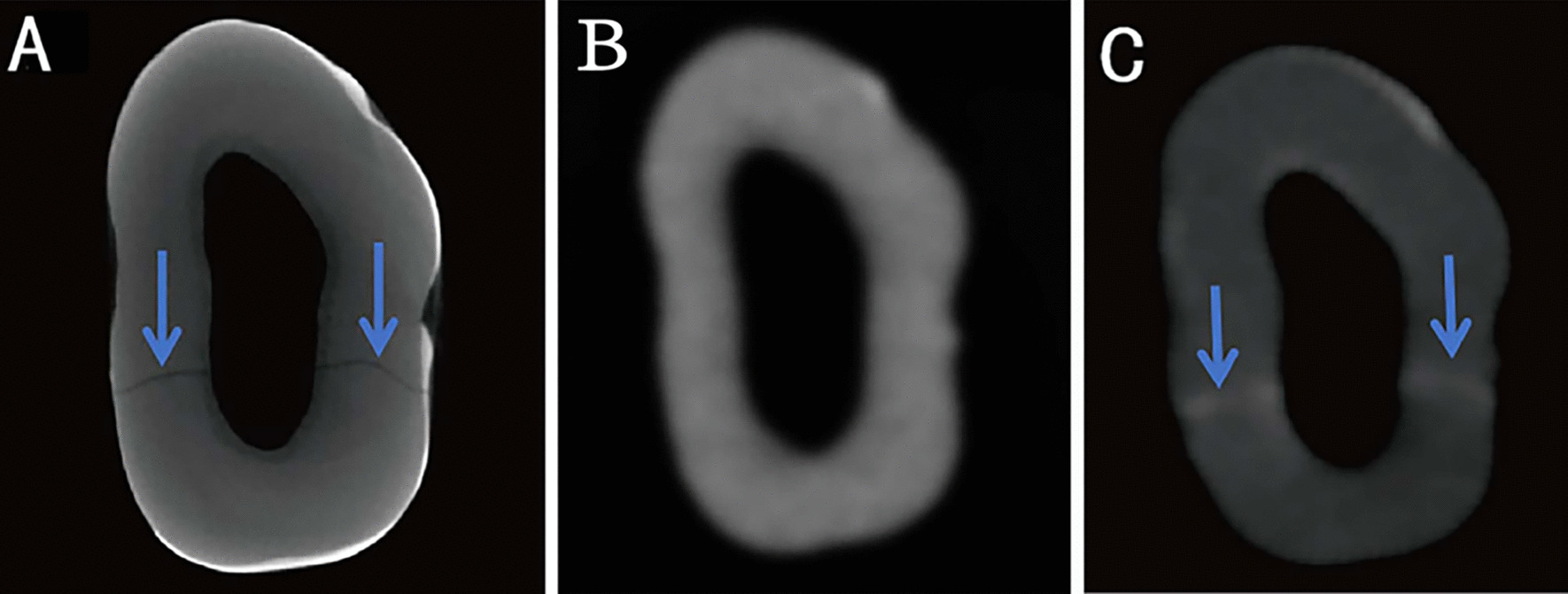
Pre-experimental cone beam computed tomography (pre-CBCT), micro-computed tomography (micro-CT), and contrast-enhanced cone beam computed tomography (contrast-enhanced CBCT) images of cracked tooth no. 8, which has a mesiodistal crack. **A** Horizontal plane of the crack on micro-CT (the blue arrows indicate the crack). **B** Horizontal plane of the crack on pre-CBCT (showing no crack). **C** Horizontal plane of the crack on contrast-enhanced CBCT (the blue arrow indicates the crack)

**Fig. 5:**
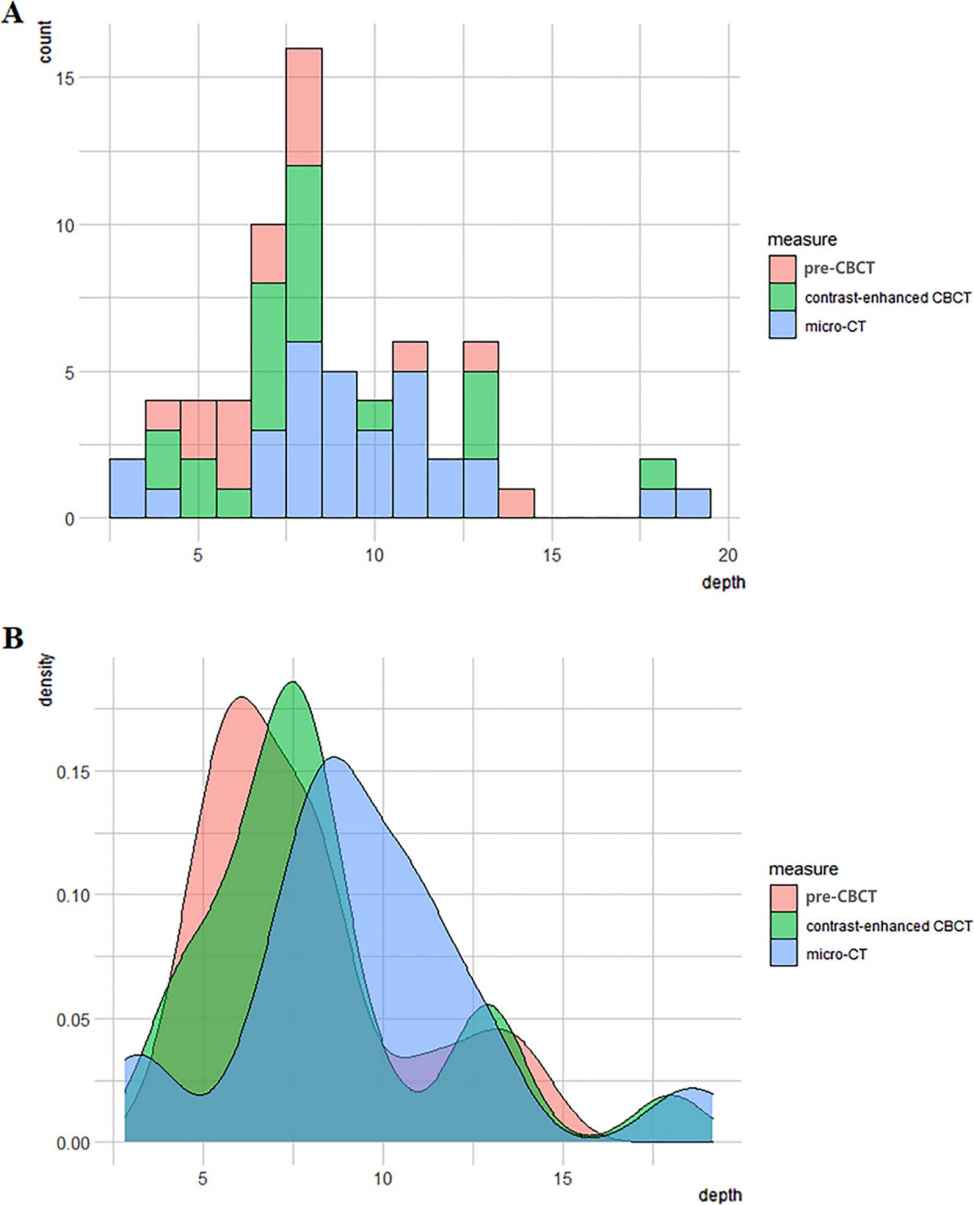
**A** The histogram of crack depths measured by pre-CBCT, contrasted-enhanced CBCT and micro-CT. **B** The kernel density plot of crack depths measured by pre-CBCT, contrasted-enhanced CBCT and micro-CT. CBCT: cone beam computed tomography; Pre-CBCT: pre-experimental cone beam computed tomography; micro-CT: micro-computed tomography

The results of the pre-CBCT, contrast-enhanced CBCT, and micro-CT were recorded (Fig. 4A–C). Thirty-one cracked teeth established by the artificial simulation model were included in this study. Of the total number of teeth, only 15 cracked teeth could be identified using pre-CBCT, 21 could be identified using contrast-enhanced CBCT, whereas 11 could be identified using both pre-CBCT and contrast-enhanced CBCT (Fig. 5A).

The sensitivity values of pre-CBCT and contrast-enhanced CBCT for diagnosing cracked teeth were 48.4%, and 67.7%, respectively, implying that the sensitivity of contrast-enhanced CBCT was higher than that of pre-CBCT (Fig. 5B).

The mean crack depth of the 10 teeth that were only measured on contrast-enhanced CBCT, but not visible on pre-CBCT was 7.114 ± 1.587 mm. Meanwhile, the mean crack depth of the 4 cracked teeth detected only during pre-CBCT, and not during contrast-enhanced CBCT, was 7.2650 ± 1.135 mm.

**Fig. 6:**
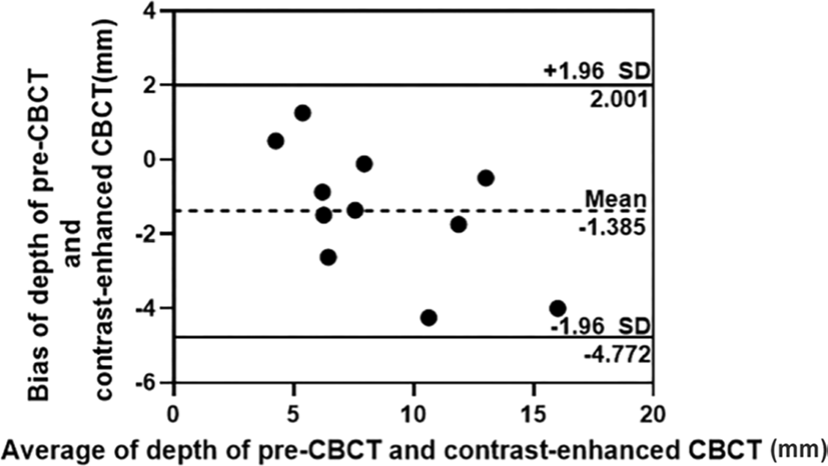
Bland–Altman plot of the crack depths on 11 cracked teeth measured both by pre-CBCT and contrasted-enhanced CBCT, with the representation of the limits of agreement, from − 1.96* s* to + 1.96* s.* Pre-CBCT: pre-experimental cone beam computed tomography

The K-means clustering algorithm was used to transform the crack depths measured by micro-CT from continuous variables into two categories, defined as deep cracks and shallow cracks. The ICC value of crack depth, measured using pre-CBCT and contrast-enhanced CBCT, was 0.847 (95% confidence interval [CI] 0.380–0.960; *P* < 0.001), demonstrating strong consistency. Both pre-CBCT and contrast-enhanced CBCT exhibited good consistency in determining the crack depth (Fig. 6).

**Fig. 7:**
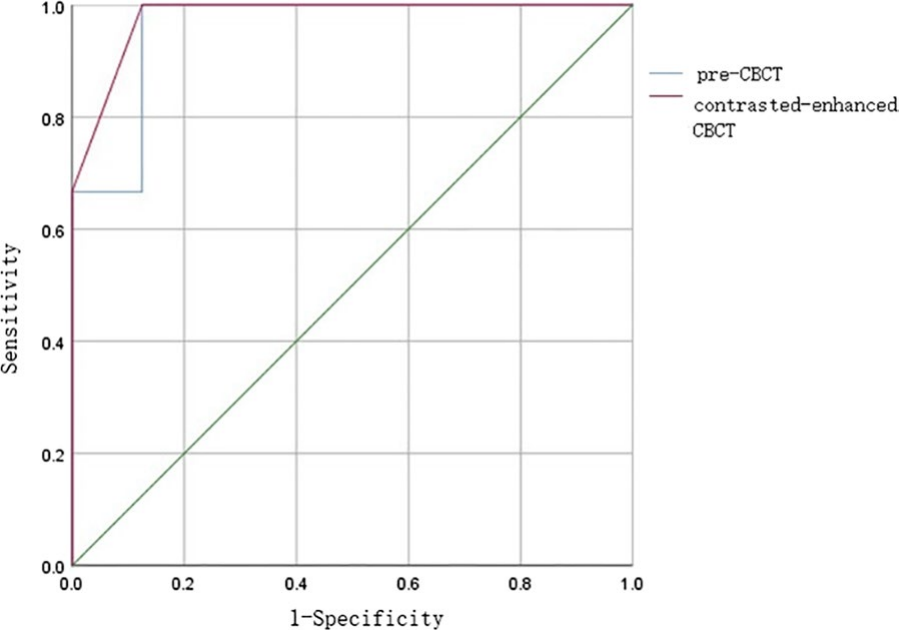
Receiver operating characteristic (ROC) curves of the two measurements. The blue line represents pre-CBCT, which had good discriminative power with area under the ROC curve (95% confidence interval) of 0.958 (95% CI 0.843–1.074). The red line represents the contrast-enhanced CBCT which had good discriminative power with area under ROC curve (95% confidence interval) of 0.979 (95% CI 0.921–1.037). Pre-CBCT: pre-experimental cone beam computed tomography

The area under the ROC curve (AUC) of pre-CBCT was 0.958 (P < 0.001, 95% CI 0.843–1.074), and that of contrast-enhanced CBCT was 0.979 (P = 0.000, 95% CI 0.921–1.037). The AUCs of pre- and contrast-enhanced CBCT were different; however, the difference was not statistically significant (Z = − 0.707, P = 0.480; Fig. 7).

**Fig. 8:**
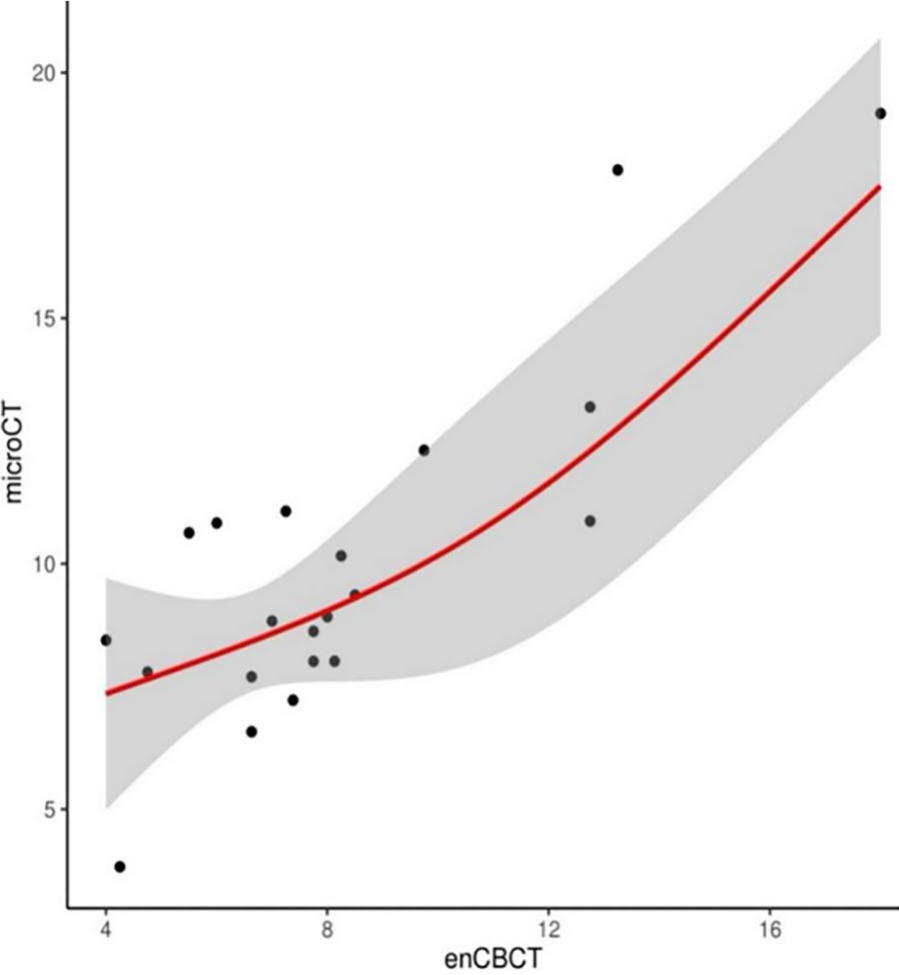
Risk-adjusted, restricted cubic splines with 4 knots of crack depths measured by contrast-enhanced CBCT and crack depths measured by micro-CT. The X axis shows the crack depths measured using contrast-enhanced CBCT; the Y axis shows the crack depths measured using micro-CT. Red line represents crack depths measured using micro-CT; Grey area represents a 95% confident interval. The root mean square error (RMSE) was 1.813, R^2^ was 0.724, *P* = 0.000. Micro-CT: micro-computed tomography; contrast-enhanced CBCT: contrast-enhanced cone beam computed tomography

The ICC value of crack depth, as measured by contrast-enhanced CBCT and micro-CT, was 0.753 (95% CI 0.248–0.911; *P* < 0.001), demonstrating strong consistency. Contrast-enhanced CBCT can explain the 72.36% of micro-CT changes, and the root mean square error was only 1.81. We observed a linear relationship between the crack depths measured by contrast-enhanced CBCT and micro-CT (Fig. 8).

## Discussion

Methylene blue staining [7, 9] performed on the tooth surface can help in the visualization of cracks, but not in the measurement of their depth. In this study, ioversol solution was used as a crack indicator via infiltration of the crack under vacuum conditions to obtain a high-density linear image using CBCT, thus allowing for imaging by contrast-enhanced CBCT and observation of the crack. Ioversol is a nonionic contrast agent, often used in angiography of the cerebral artery, coronary artery, and peripheral artery, as well as in other types of angiography [36, 37]. It helps obtain high-density images by radiography, thus allowing clear imaging of the vascular path. In this study, it was used as a "dye" to explore whether it can display cracks on contrast-enhanced CBCT images.

Our results indicate that contrast-enhanced CBCT has high sensitivity. The X-ray photons passing through a radiolucent fracture plane also pass through extensive amounts of radiopaque healthy tooth structure; thus, tooth cracks may not be apparent [38]. Therefore, if measures were taken so that the crack image reveals higher density than that of healthy structure, it is more likely that the crack will not be masked by healthy tooth structure. Ioversol as a medium enables high-resolution morphological assessment of articular cartilage in small animals using CBCT [39]. It has been inferred that ioversol could be used in hard tissue imaging to enhance the diagnostic ability. A recent study has shown that contrast-enhanced CBCT using meglumine iatrizoate as a medium can reveal greater number of crack lines [40].

The fact that the 4 teeth detected only by pre-CBCT had a greater mean crack depth than that of the 10 teeth only observable on contrast-enhanced CBCT might be explained by the following: (i) the crack direction of the artificial model in this study is uncertain, making ioversol infiltration difficult; (ii) the crack depth in the present study was not controlled, and the above two sets of data are not comparable.

Our study revealed no significant differences between pre-CBCT and contrast-enhanced CBCT in measuring the crack depth of cracked teeth. This may be because crack width impacts the diagnostic accuracy of CBCT in detecting cracked teeth [41–43]. If the crack is wide, the results of pre-CBCT and contrast-enhanced CBCT may not differ significantly. However, when the crack is narrow, it may not be clearly visible during pre-CBCT, but can be seen more clearly on contrast-enhanced CBCT when ioversol is used as a medium. Therefore, when cracks can definitively be detected on pre-CBCT, their depths (when measured using pre-CBCT) are very close to those measured using contrast-enhanced CBCT.

Furthermore, the crack depths measured on contrast-enhanced CBCT and micro-CT demonstrated good consistency. The result of RCS revealed that the relationship between the difference in the crack depths measured on contrast-enhanced CBCT and micro-CT to be linear. However, a formula cannot be obtained to calculate the specific relationship between the depth measured by contrast-enhanced CBCT and micro-CT.

It is worth mentioning that the crack depth measured by both micro-CT and contrast-enhanced CBCT was a vertical distance from the crown to the root, i.e., it was not the actual length of the crack. Considering that the crack line passing through the three different dimensions may exhibit inconsistencies and that most cracks were oblique, it was difficult to access the actual full length of the crack in a single plane. Clinically, the prognosis for teeth with cracks that extend vertically through the pulp, or involve the subpulpal floor, is less favorable [44]. Therefore, in future studies, the actual length of the crack and the crack path should be studied by developing 3D models of cracks using different software. Additionally, a narrow and deep periodontal probing depth means that the crack has progressed deep into the root [45]. The crack extension depth from the crown to the root on the mesial or distal surface should be given more attention, as the degree of damage to the full length of the tooth can be evaluated better when the cracked tooth involves one or both marginal ridges. Therefore, future research on structural cracks causing clinical symptoms will have more clinical significance.

There are some limitations to our study: (i) the sample size was small. Thus, the results need to be further confirmed using a larger sample size; (ii) the study was conducted in vitro; thus, the feasibility of the clinical application of this technique needs to be confirmed in the future; and (iii) the diagnostic capacity for the crack-width axis of the infiltrated contrast agent should be analyzed in future studies.

We conducted this study and aimed to develop a new technique based on CBCT to improve the detection of cracked teeth and the accuracy of crack depth evaluation in vitro. The study suggests that CBCT with a high-density contrast medium is reliable for detecting cracks. On the basis of our findings, simulated animal experiments in controlled negative pressure conditions are warranted. Moreover, if this method is viable, we might have resolved the challenges faced while managing cracked teeth.

## Conclusion

Contrast-enhanced CBCT under vacuum conditions with a contrast medium can significantly improve the crack detection rate of cracked teeth; however, it cannot measure the crack depth accurately.

## Data Availability

The datasets used and/or analyzed during the current study are available from the corresponding author on reasonable request.

## Abbreviations

CBCT: Cone beam computed tomography
pre-CBCT: Pre-experimental CBCT
micro-CT: Micro-computed tomography
OCT: Optical coherence tomography
QLF: Quantitative laser-induced fluorescence
VRFs: Vertical root fractures
MRI: Magnetic resonance imaging
ICC: The intraclass correlation coefficient
ROC: Receiver operating characteristic
AUC: The areas under the ROC curves
RCS: Restricted cubic splines
